# Comparative antibacterial activities of neutral electrolyzed oxidizing water and other chlorine-based sanitizers

**DOI:** 10.1038/s41598-019-56248-7

**Published:** 2019-12-27

**Authors:** Abiodun D. Ogunniyi, Catherine E. Dandie, Sergio Ferro, Barbara Hall, Barbara Drigo, Gianluca Brunetti, Henrietta Venter, Baden Myers, Permal Deo, Erica Donner, Enzo Lombi

**Affiliations:** 1 Future Industries Institute, University of South Australia, Mawson Lakes, South Australia Australia; 2 Ecas4 Australia Pty Ltd, 8/1 London Road, Mile End South, South Australia Australia; 3 Plant Health and Biosecurity, SARDI, Adelaide, South Australia Australia; 4 School of Pharmacy and Medical Sciences, University of South Australia, Adelaide, South Australia Australia; 5 Natural and Built Environments Research Centre, School of Natural and Built Environments, University of South Australia, Mawson Lakes, South Australia Australia

**Keywords:** Hydrology, Water microbiology

## Abstract

There is increasing demand for safe and effective sanitizers for irrigation water disinfection to prevent transmission of foodborne pathogens to fresh produce. Here we compared the efficacy of pH-neutral electrolyzed oxidizing water (EOW), sodium hypochlorite (NaClO) and chlorine dioxide (ClO_2_) against single and mixed populations of *E. coli*, *Listeria* and *Salmonella* under a range of pH and organic matter content. EOW treatment of the mixed bacterial suspension resulted in a dose-dependent (<1 mg/L free chlorine), rapid (<2 min) and effective (4–6 Log_10_) reduction of the microbial load in water devoid of organic matter under the range of pH conditions tested (pH, 6.0, 7.0, 8.4 and 9.2). The efficacy of EOW containing 5 mg/L free chlorine was unaffected by increasing organic matter, and compared favourably with equivalent concentrations of NaClO and ClO_2_. EOW at 20 mg/L free chlorine was more effective than NaClO and ClO_2_ in reducing bacterial populations in the presence of high (20–100 mg/L) dissolved organic carbon, and no regrowth or metabolic activity was observed for EOW-treated bacteria at this concentration upon reculturing in rich media. Thus, EOW is as effective or more effective than other common chlorine-based sanitizers for pathogen reduction in contaminated water. EOW’s other characteristics, such as neutral pH and ease of handling, indicate its suitability for fresh produce sanitation.

## Introduction

Microbial contamination of fresh produce such as spinach, lettuce, parsley and other leafy greens by opportunistic and human pathogens continues to be a major source of foodborne illnesses and disease outbreaks worldwide^1^. In most instances, pre-harvest water such as irrigation water and post-harvest washing water have been identified as the main sources of contamination associated with human illness^2,3^. Current water disinfection processes involve either the use of chemicals (such as chlorine, ozone, peracetic acid, or hydrogen peroxide), or non-chemical disinfection methods such as ultraviolet irradiation and membrane filtration^3–9^. However, these treatment technologies all have shortcomings in terms of efficacy and/or safety concerns. Consequently, there is a growing global focus on the deployment of safe, effective and environmentally-sustainable irrigation water and post-harvest sanitation technologies. One approach being explored is the use of electrolyzed oxidizing water (EOW), which is generated through the electrolysis of chloride-containing water (generally in the form of sodium or potassium chloride (NaCl/KCl) to form hypochlorous acid and reactive oxygen species (^**∙**^OH, O_3_, H_2_O_2_) that are toxic to microorganisms^10,11^. The various types of EOW described in the literature include acidic EOW (pH 2–3), slightly acidic EOW (pH 5–6.5), alkaline EOW (pH 10–13), slightly alkaline EOW (pH 8–10), and neutral EOW (pH 7–8)^12^.

Studies investigating EOW treatment of aqueous human pathogen suspensions under varying conditions of exposure time, pH, temperature, available chlorine, and redox potential have consistently shown substantial log reductions in viable microorganisms^13–16^. Of the various types of EOW, neutral EOW has been considered the most promising as it contains predominantly HOCl, which is more effective than ClO^−^ for microbial cell wall penetration and oxidative attack^12,17^. However, there are limited published applications of neutral EOW use in the irrigation and washing of fresh vegetables^18,19^ or fruit^20,21^, with publications to date mainly focussing on its use in the seafood^22^ and meat^17,23,24^ industries. The use of Na-based salts rather than K for generation of the EOW might be of concern in the context of vegetable production, because of the potential problems associated with Na accumulation in soil, in contrast to the potential benefit of K supplementation for crop growth.

In this study, we aimed to establish boundary conditions (in terms of pH and organic matter content) for the effective use of neutral EOW prepared using either Na or K salts. Organic matter was introduced in the form of purified natural organic matter, γ-sterilized manure, and in filter-sterilized secondary and tertiary treated wastewater. We also compared EOW efficacy against surrogate foodborne pathogens with that of other chlorine-based sanitizers (sodium hypochlorite and chlorine dioxide). We hypothesized that EOW treatment could significantly reduce the microbial load in contaminated water, thereby expanding the range of safe source water options for fresh produce irrigation. We further hypothesized that the efficacy of EOW to reduce the microbial load would be at least comparable to that of the other chlorine-based sanitizers and that its efficacy would not be diminished under a range of pH conditions but might be under conditions of high organic matter content. Finally, we investigated the efficacy of EOW and other chlorine-based sanitizers in relation to their potential to induce viable but nonculturable (VBNC) cells. This is a significant concern associated with disinfection processes, especially in terms of the potential dissemination of VBNC pathogens via treated irrigation or post-harvest wash water. The VBNC state is a survival strategy used by many bacteria in response to adverse environmental conditions^25,26^ and multiple works have described the potential for induction of the VBNC state during water disinfection processes^27–29^. This is of importance for improving the quality and safety of fresh produce and preventing future outbreaks, thereby increasing consumer confidence in consumption, particularly by vulnerable individuals.

## Methods

### Bacterial strains, growth conditions and inocula preparation

The bacterial strains used in this study were *Escherichia coli* (ATCC 25922), bioluminescent *E. coli* WS2572 (Xen14), *Listeria innocua* 6a (ATCC 33090) and *Salmonella enterica* serovar Enteritidis 11RX^30,31^. Glycerol stock cultures were maintained at −80 °C and were streaked onto Luria Bertani (LB) agar (Oxoid; Thermo Fisher Scientific, Scoresby, VIC, Australia) to obtain isolated colonies. Single colonies were streaked onto the following selective agar plates (Thermo Fisher Scientific) for presumptive identification: Eosin Methylene Blue agar (EMB; PP2169) for *E. coli*, Oxford *Listeria* Selective agar (OXF; PP2141) for *L. innocua* 6a, and Xylose Lysine Deoxycholate agar (XLD; PP2004) for *S*. Enteritidis 11RX.

For experiments, single colonies from selective agar plates were emulsified in LB broth and grown overnight at 37 °C with aeration at 150 rpm on a digital platform mixer (Ratek Instruments, Boronia, VIC, Australia). Thereafter, bacteria were subcultured at a 1:10 dilution into fresh LB broth and incubated further at 180 rpm until the optical density at 600 nm (*OD*_600_) = 1.0 (for *E. coli* and *S*. Enteritidis 11RX) or *OD*_600_ = 0.5 (for *L. innocua* 6a) was reached (equivalent to approx. 1 × 10^9^ colony-forming units (CFU)/mL for each strain). Bacteria were then harvested and washed extensively (3×) in autoclave-sterilized Milli-Q water (PURELAB Classic, ELGA; High Wycombe, UK) to remove residual culture medium and then resuspended in sterile Milli-Q water to approx. 1 × 10^9^ CFU/mL for each strain. Where mixed bacterial suspensions were tested, the bacteria were mixed immediately prior to use at approximately equal concentrations to ensure that there was no substantial change in the relationship among the bacteria in the time between mixing and application of the disinfection treatments. The initial concentration of bacteria was high (~2 × 10^8^ CFU/mL) to simulate the worst-case scenario of high bacterial load.

### Reagents, solutions and instruments

Neutral EOW was provided by Ecas4 Australia Pty Ltd (8/1 London Road, Mile End South, SA, Australia) at 300–350 mg/L free chlorine. Sodium hypochlorite (NaClO; UN No 1791) was obtained as a 12.5% solution from Chemwell Pty Ltd (3 Clive St, Springvale, VIC, Australia). Chlorine dioxide (ClO_2_) was obtained as TwinOxide Tabs® Part No 121710 (TwinOxide®; supplied by Integra Water, Regency Park, SA, Australia) and was prepared as a 1,000 mg/L chlorine solution according to the manufacturer’s instructions. Several different treatment solutions were established to test the efficacy of EOW, NaClO and ClO_2_. Suwannee River natural organic matter (SRNOM; 2R101N, International Humic Substances Society, St Paul, MN, USA) was used as the source of natural organic matter and was resuspended to the equivalent of 200 mg/L dissolved organic carbon (DOC). Cow manure was γ-sterilized at Steritech (Melbourne, VIC, Australia), dried in an oven at 37 °C and ground to a fine powder using an analytical mill (IKA, Selangor, Malaysia) and then resuspended to the equivalent of 10 g/L in sterile Milli-Q water (pH 7.0). Finally, secondary treated wastewater and tertiary treated effluent from a wastewater treatment plant in Adelaide, South Australia were filter sterilized to produce test solutions with realistic background chemistry. The DOC contents in the SRNOM suspension, cow manure suspension and secondary and tertiary treated effluents were measured on a Shimadzu TOC-L total organic carbon analyzer (Shimadzu Australasia, Rydalmere, NSW, Australia).

Milli-Q water at pH 6.0, 7.0, or 8.4 was prepared using 1 mM NaNO_3_ as background electrolyte, while Milli-Q water at pH 9.2 was prepared in 0.01 M carbonate-bicarbonate buffer (9.1 mM sodium bicarbonate and 0.9 mM anhydrous sodium carbonate) and verified on a Eutech Instruments PC 700 pH meter (Thermo Fisher Scientific). Dilutions of disinfectants with different estimated free chlorine concentrations were prepared in these buffers. The amount of free chlorine in EOW and NaClO was measured using a free chlorine and chlorine ultra-high range portable photometer (HI 96771 C; Hanna Instruments, Keysborough, VIC, Australia) according to the manufacturer’s recommendations, while the concentration of ClO_2_ was measured using method 10126 for water on a HACH DR/890 Colorimeter (Hach Pacific, Dandenong South, VIC, Australia) according to the manufacturer’s instructions.

### Time-kill and dose response analysis of EOW

#### Time kill

A time-kill experiment was conducted to determine the appropriate length of time required for bacterial inactivation by EOW. Na-based EOW was prepared to provide 0, 3, 30 and 150 mg/L free chlorine in Milli-Q water (pH 7.0), to each of which approx. 2 × 10^8^ CFU *E. coli*, *L. innocua* 6a or *S*. Enteritidis 11RX was added. Aliquots were then withdrawn at 0, 5, 30, 60, 120, 300 and 600 s and disinfectant activity was neutralized with a 0.05% (v/v) final concentration of sodium thiosulphate (Na_2_S_2_O_3_). Viable counts were obtained by serial 10-fold dilution in phosphate-buffered saline (PBS) and plating onto selective (OXF for *L. innocua*, EMB for *E. coli* and XLD for *S*. Enteritidis) and non-selective (LB for all strains) media followed by incubation overnight at 37 °C. The limit of detection for viable counts was set at 100 CFU/mL in all experiments. All experiments were independently repeated four times.

#### Comparison of Na- and K-based EOW and dose-response assessments

From the time-kill experiment, a time point of 120 s was chosen for all subsequent experiments as a reasonable worst-case scenario of a short contact time under field conditions. A comparison of the efficacy of Na- or K-based EOW was conducted at a range of chlorine concentrations up to 4.8 mg/L followed by a dose-response experiment with the mixed bacterial suspension using the Na-based EOW. Disinfectant inactivation, dilution and plating were conducted as described above.

### Effect of pH on EOW efficacy and comparison with other sanitizers (NaClO and ClO_2_)

The efficacy of EOW was tested at pH 6.0, 7.0 and 8.4 (unbuffered) or at pH 6.0, 7.0 and 9.2 (buffered) at free chlorine concentrations of up to 4.8 mg/L for the mixed culture as described above. Based on the above experiment, the efficacy of EOW was compared with that of NaClO and ClO_2_ at equivalent free chlorine concentrations of up to 4.8 mg/L at pH 7.0. Disinfectant inactivation, dilution and plating were conducted as described above.

### Effect of organic matter content on EOW efficacy and comparison with NaClO

The effect of increasing concentrations of DOC on the amount of free chlorine present in EOW that had been prepared with initial concentrations of 1 and 5 mg/L free chlorine was determined using SRNOM concentrations of up to 40 mg/L DOC. The abilities of EOW and NaClO to reduce microbial loading in the presence of organic matter were compared using EOW and NaClO at 1 and 5 mg/L free chlorine concentration in the presence of SRNOM at concentrations of up to 40 mg/L DOC. The viability assays were conducted on the mixed bacterial culture as described above. The cells were added to organic matter solutions prior to initiation of the timed assay by the addition of the sanitizer solution.

### Comparative assessment of the bactericidal action of EOW, NaClO, and ClO_2_

To investigate the potential for induction of the viable but nonculturable (VBNC) state by the different sanitizers, a combination of metabolic activity measurements and molecular approaches were used.

For metabolic activity measurements, ~5 × 10^7^ CFU of bioluminescent *E. coli* Xen14 (PerkinElmer Inc, MA, USA) was added to EOW, NaClO or ClO_2_ prepared at 0, 1, 5, 20 and 50 mg/L free chlorine in the presence of either 40 mg/L DOC from SRNOM or 100 mg/L DOC from cow manure for 120 s at room temperature, before stopping the reaction using Na_2_S_2_O_3_. Untreated bacteria resuspended in sterile RO water, 40 mg/L DOC from SRNOM or 100 mg/L DOC from cow manure were used as controls. Samples were then serially diluted in PBS and plated on LB agar for bacterial enumeration. To measure bioluminescence, ~1 × 10^6^ CFU of each treatment was added to 200 µL sterile LB broth in a Nunc™ F96 MicroWell™ Black plate (Thermo Scientific, 237105) which was then incubated at 37 °C in a Cytation 5 Cell Imaging Multi-Mode Reader (BioTek; Winooski, VT, USA). Total luminescent signals (relative light units) and optical density measurements (*A*_600nm_) were collected over a 40-h incubation period. In another set of experiments, sterile RO water, tertiary treated wastewater (containing 5.6 mg/L DOC) or secondary treated wastewater (containing 19.2 mg/L DOC) were treated as described above. Each experiment was performed on two separate occasions. On one occasion, 20 µL samples from the experiment using the secondary and tertiary treated effluents after 40 h incubation were re-inoculated into 180 µL sterile LB broth in a Nunc™ F96 MicroWell™ Black plate and incubated at 37 °C for an additional 40 h in the Cytation 5 Cell Imaging Multi-Mode Reader to examine any potential regrowth or metabolic activity.

For the molecular analysis of the luminescent *E. coli* (Xen14) cell populations treated above, the propidium monoazide (PMAxx™) real-time PCR bacterial viability protocol (Biotium, USA; Cat No 31050-X) was used, following the manufacturer’s recommendations, essentially as described recently^32^ using the PMA Enhancer solution for Gram-negative bacteria and PMA-Lite™ LED Photolysis Device for photoactivation. Genomic DNA from bacteria treated above was extracted using the QiIAamp DNA Mini kit (Cat No: 51304; QIAGEN) following the protocol for DNA extraction from Gram-negative bacteria. Quantitative PCR was performed on a LC480 II instrument (Roche Diagnostics) using 16 S rRNA gene primers F: 5′-TCCTACGGGAGGCAGCAGT-3′ and R: 5′-ATTACCGCGGCTGCTGG–3′ and associated fast cycling parameters in Cat No 31050-X (Biotium).

## Results and Discussion

### Neutral EOW elicits a rapid, dose-dependent and substantial reduction in viable counts of single or mixed bacterial suspensions in water

Results of preliminary experiments showed that EOW at 3 mg/L consistently inhibited the growth of the tested bacteria at 120 s post-exposure (not shown). Non-linear regression analysis indicated that the EOW treatment of the mixed bacterial suspension at low doses (<1 mg/L free chlorine) resulted in substantial (4–6 Log_10_) reduction in viable counts of all the bacteria tested, comparable to that reported for similar sanitizers by other researchers (reviewed by Rahman *et al*.^12^).

### Sodium (Na)- and potassium (K)-based EOW elicit similar efficacy profiles

**Figure 1:**
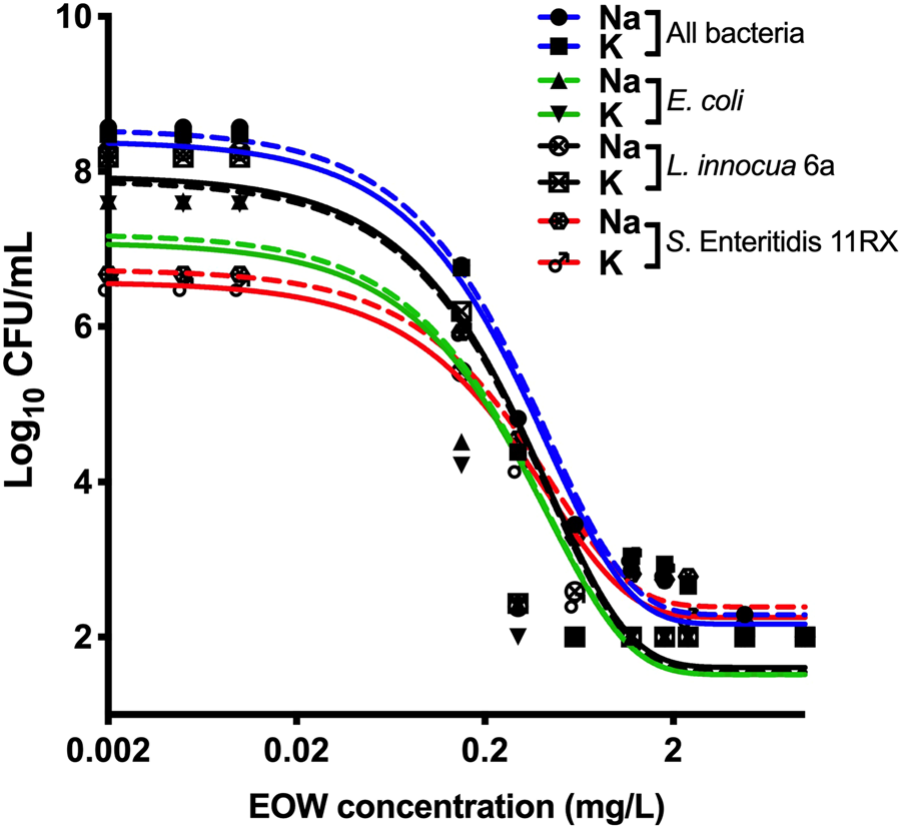
Comparison of sodium (Na)- and potassium (K)-based EOW against a mixed bacterial suspension of *Escherichia coli*, *Listeria innocua* 6a and *Salmonella* Enteritidis 11RX. EOW: electrolyzed oxidizing water in mg/L of free chlorine; CFU: colony forming units. Figures were generated using Prism v8 (GraphPad Software, San Diego, CA, USA).

The ability of Na- and K-based EOW to inhibit bacterial growth was compared to determine whether there are differences in their efficacy, using the time and dose-dependent kill kinetics of the Na-based EOW established above. Our results showed that the efficacy kinetics of Na- and K-based EOW were remarkably similar (Fig. 1). This could be valuable information for growers who might be concerned about Na levels in irrigation water and would prefer to use K-based EOW instead.

### EOW is effective under a range of pH conditions

**Figure 2:**
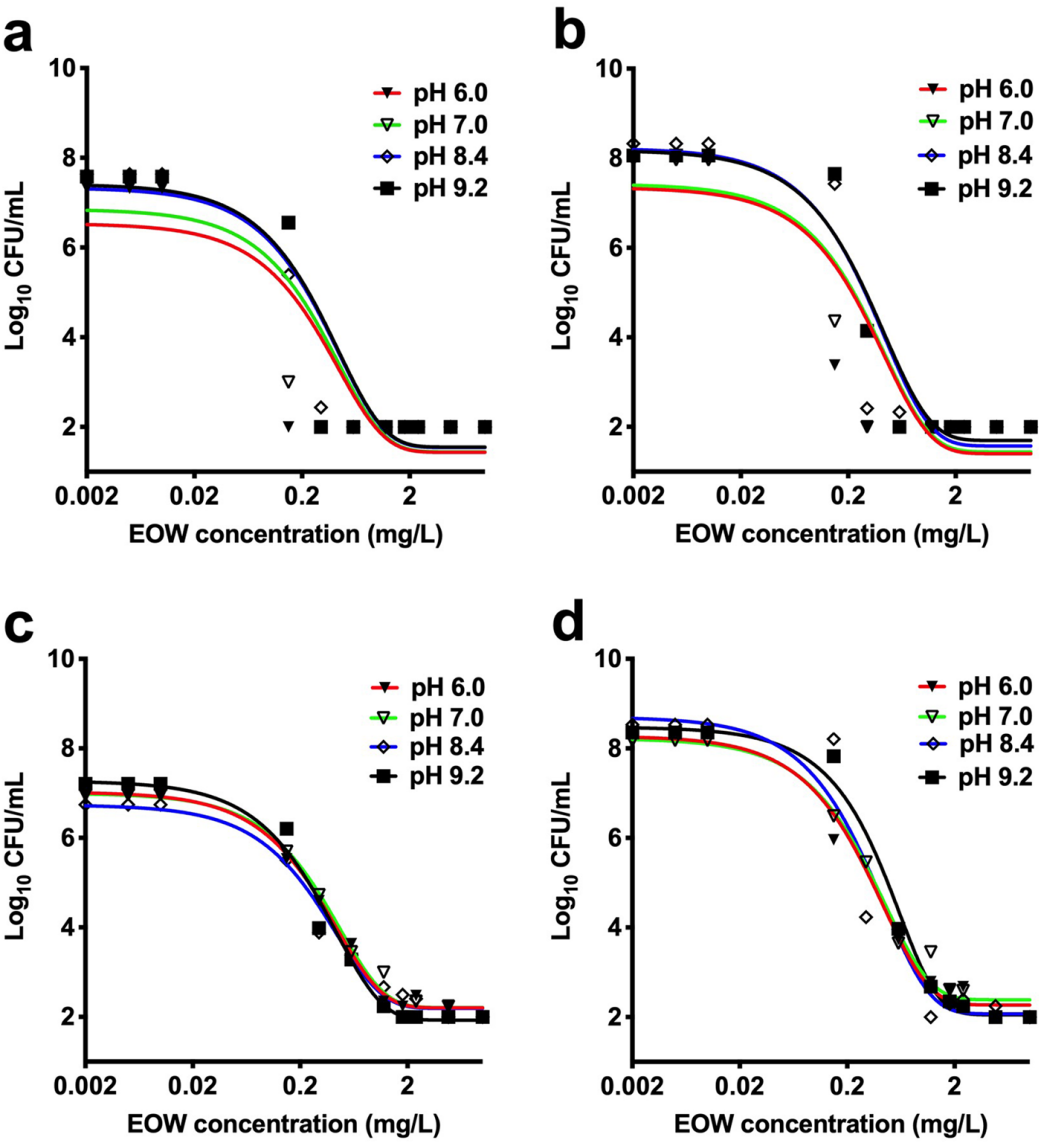
Bactericidal activity of electrolyzed oxidizing water (EOW) under a range of pH conditions. (**a**) *Escherichia coli*, (**b**) *Listeria innocua* 6a, (**c**) *Salmonella* Enteritidis 11RX, and (**d**) mixed bacterial culture. CFU: colony forming units; EOW concentration refers to mg/L of free chlorine. Figures were generated using Prism v8 (GraphPad Software, San Diego, CA, USA).

The efficacy of EOW was tested against the mixed bacterial population at pH 6.0, 7.0, or 8.4 (using 1 mM NaNO_3_ as background electrolyte). We found that the NaNO_3_ electrolyte did not function as an effective buffer, with the pH decreasing to near neutral for the pH 8.4 solution on addition of increasing concentrations of EOW. Thereafter, water at pH 9.2 was prepared in 0.01 M carbonate-bicarbonate buffer, which was stable in the presence of increasing EOW concentration, and the experiment was repeated. Overall, our results showed that the activity and efficacy of the EOW was not appreciably affected under the range of unbuffered and buffered pH conditions (pH 6.0, pH 7.0, pH 8.4 and pH 9.2) tested (Fig. 2). The efficacy of EOW at pH 9.2 was somehow unexpected, as Pangloli and Hung^33^ found that the bactericidal efficacy of EOW against *E. coli* O157:H7 at pH values in the range of 5–8 was unaffected, but that there was a significant decrease in efficacy at pH 8 against *L. monocytogenes*. Similarly, Rahman *et al*.^15^ found that the ability of EOW to inactivate all organisms was diminished at pH 9.0. It is unclear which factors contributed to the high activity of the neutral EOW used in this study at high pH. However, it is possible that the consistent free chlorine content and high oxidation-reduction potential of EOW in our study might have contributed synergistically to its efficacy regardless of pH, as suggested by some studies^34,35^.

### The activity of EOW compares favourably with equivalent concentrations of other chlorine-based sanitizers (NaClO and ClO_2_)

**Figure 3:**
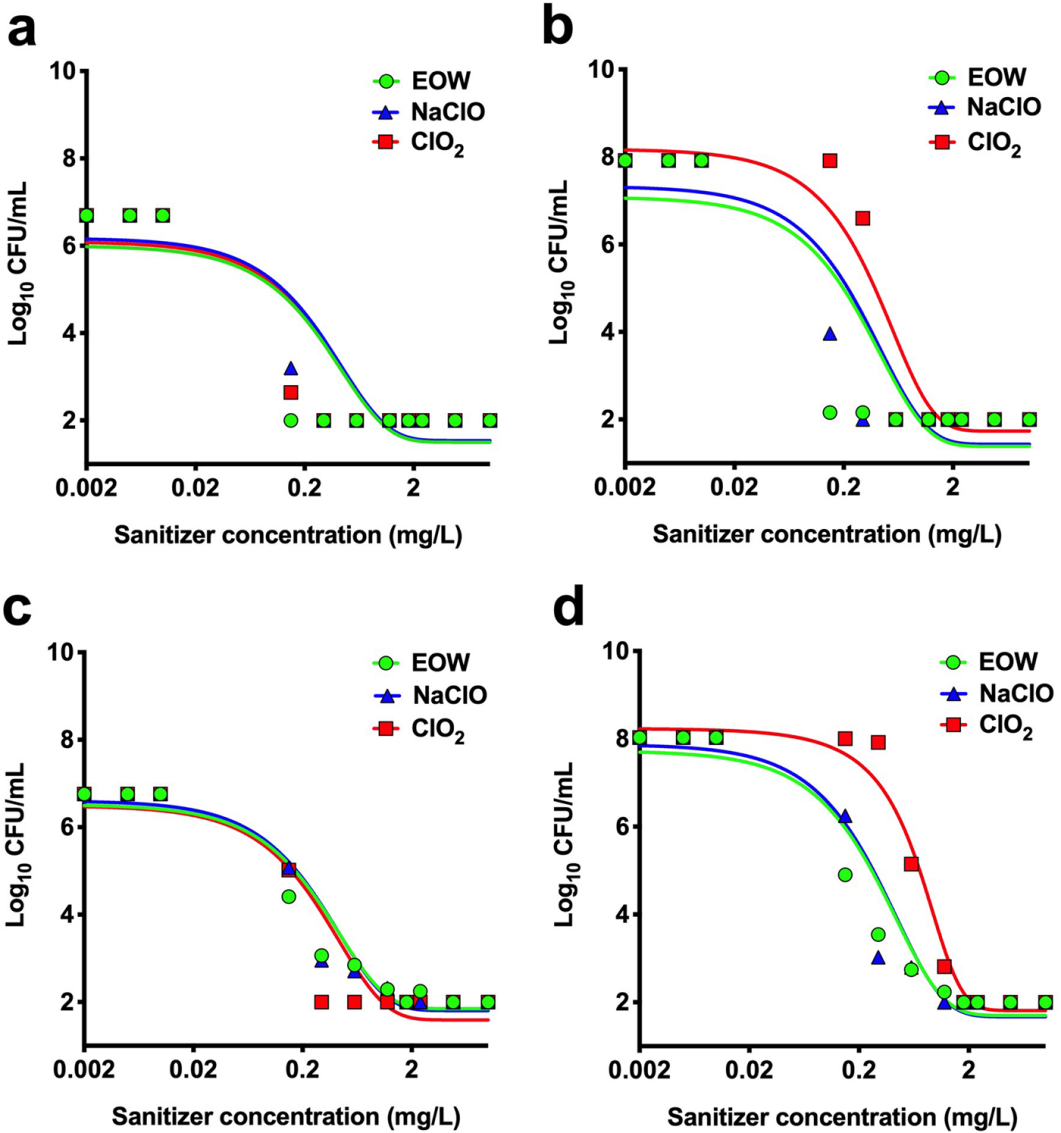
Comparison of electrolyzed oxidizing water (EOW) activity with equivalent concentrations of sodium hypochlorite (NaClO) and chlorine dioxide (ClO_2_) expressed as mg/L of free chlorine. (**a**) *Escherichia coli*, (**b**) *Listeria innocua* 6a, (**c**) *Salmonella* Enteritidis 11RX, and (**d**) mixed bacterial culture. Figures were generated using Prism v8 (GraphPad Software, San Diego, CA, USA).

We tested the hypothesis that the efficacy of EOW to reduce the microbial load is (at least) comparable to equivalent free chlorine concentrations of other chlorine-based sanitizers (NaClO and ClO_2_). Our analysis showed that the efficacy of EOW in reducing the microbial load compared favourably with that of equivalent concentrations of NaClO and ClO_2_ (Fig. 3). Our analysis confirmed the hypothesis, as NaClO and EOW treatments gave the same results, whereas ClO_2_ did not perform as well in the case of *L. innocua* and the mixed bacterial culture (Fig. 3).

### Activity of EOW in the presence of increasing organic matter content

**Figure 4:**
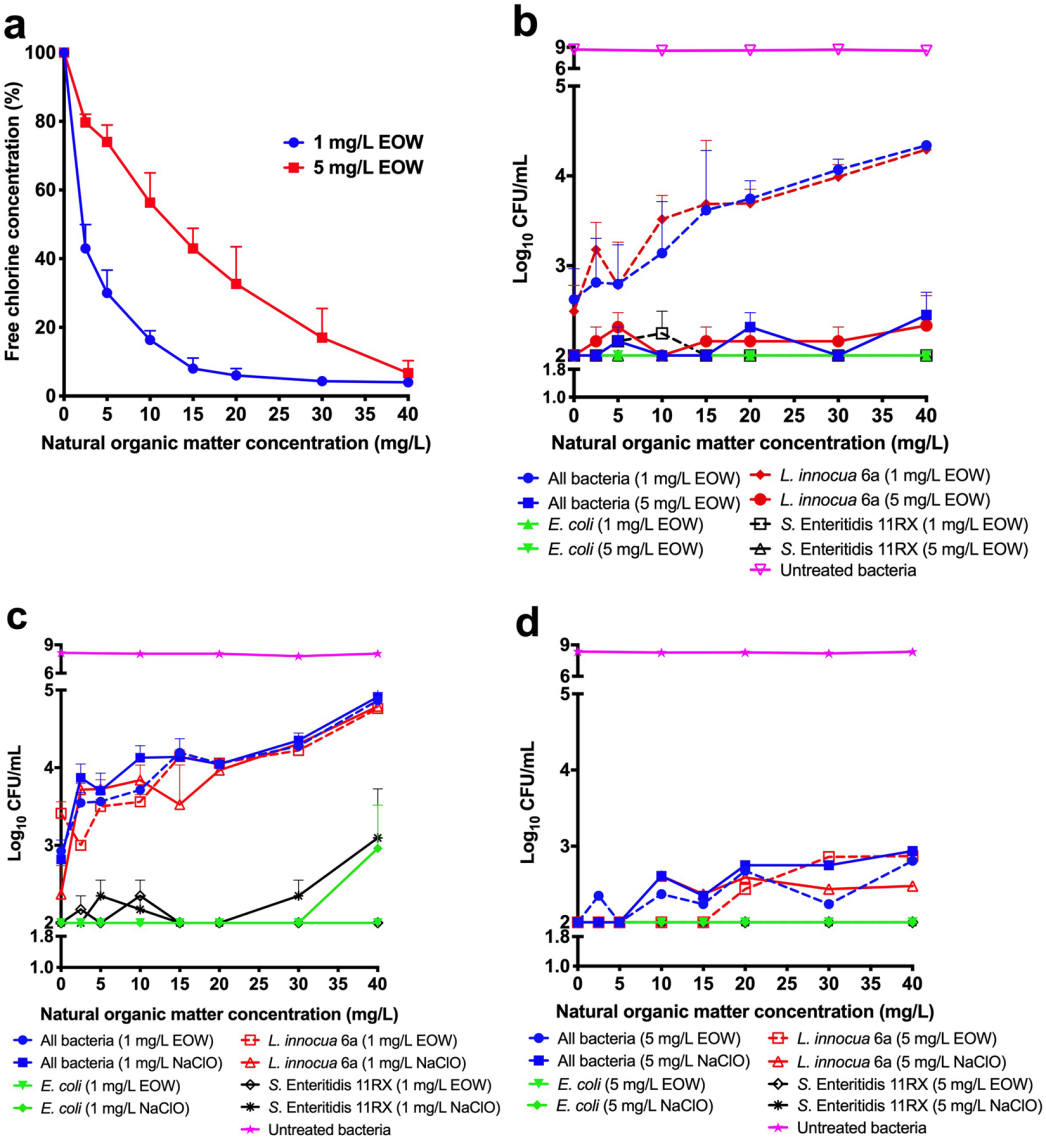
Effect of natural organic matter on activity of electrolyzed oxidizing water (EOW) and sodium hypochlorite (NaClO). (**a**) Quenching of free chlorine concentration by increasing organic matter content, (**b**) Inhibitory activity of EOW in the presence of increasing organic matter content, (**c**,**d**) Comparison of bactericidal activities of EOW and NaClO in the presence of increasing organic matter content. *E. coli*: *Escherichia coli*; *L. innocua*: *Listeria innocua*; *S*. Enteritidis: *Salmonella* Enteritidis. Figures were generated using Prism v8 (GraphPad Software, San Diego, CA, USA).

We tested the effects of increasing concentrations of DOC (using SRNOM) on the amount of free chlorine present in EOW at starting concentrations of 1 or 5 mg/L free chlorine. We found a dose-dependent reduction in the amount of free chlorine at both concentrations, with only 0.43 mg/L of free chlorine residual in the 1 mg/L EOW in the presence of 2.5 mg/L SRNOM, and 0.85 mg/L of free chlorine in the 5 mg/L EOW in the presence of 30 mg/L SRNOM (Fig. 4a).

We then tested the hypothesis that the efficacy of EOW to reduce the microbial loading of irrigation water will be reduced in the presence of organic matter by assessing the bactericidal activity of EOW in the presence of SRNOM. As expected, we found that the ability of EOW containing 1 mg/L free chlorine to reduce the bacterial population was substantially reduced in the presence of increasing organic matter content, but its efficacy was not appreciably affected when the SRNOM was added to EOW containing 5 mg/L free chlorine (Fig. 4b).

### Efficacy of EOW and NaClO are similar in the presence of organic matter

We compared the efficacy of 1 and 5 mg/L EOW and NaClO in the presence of increasing organic matter content. The efficacy of ClO_2_ was not assessed on this occasion as it did not perform as well as EOW and NaClO in our earlier evaluation. Our results show that there was progressive inactivation of both 1 mg/L EOW and 1 mg/L NaClO (Fig. 4c), as observed earlier for EOW (Fig. 4a,b) and as reported by other researchers^36,37^. The mechanism by which organic matter reduces the activity of EOW or NaClO is by quenching the activity of the free available chlorine, leading to lower concentrations of chlorine available to act on pathogens; if the concentration of free chlorine is reduced to below the effective concentration required to kill the pathogen, this might lead to reduced kill rates and/or induction of VBNC cells^38,39^ (also see below). However, both EOW and NaClO were still strongly inhibitory in the presence of increasing organic matter when their starting concentration was set at 5 mg/L free chlorine (Fig. 4d). Together, these results strongly indicate that the efficacy of EOW to reduce the microbial load is (at least) comparable to that of the other chlorine-based sanitizers.

### EOW is bactericidal and could potentially reduce induction of the VBNC state in bacterial populations

**Figure 5:**
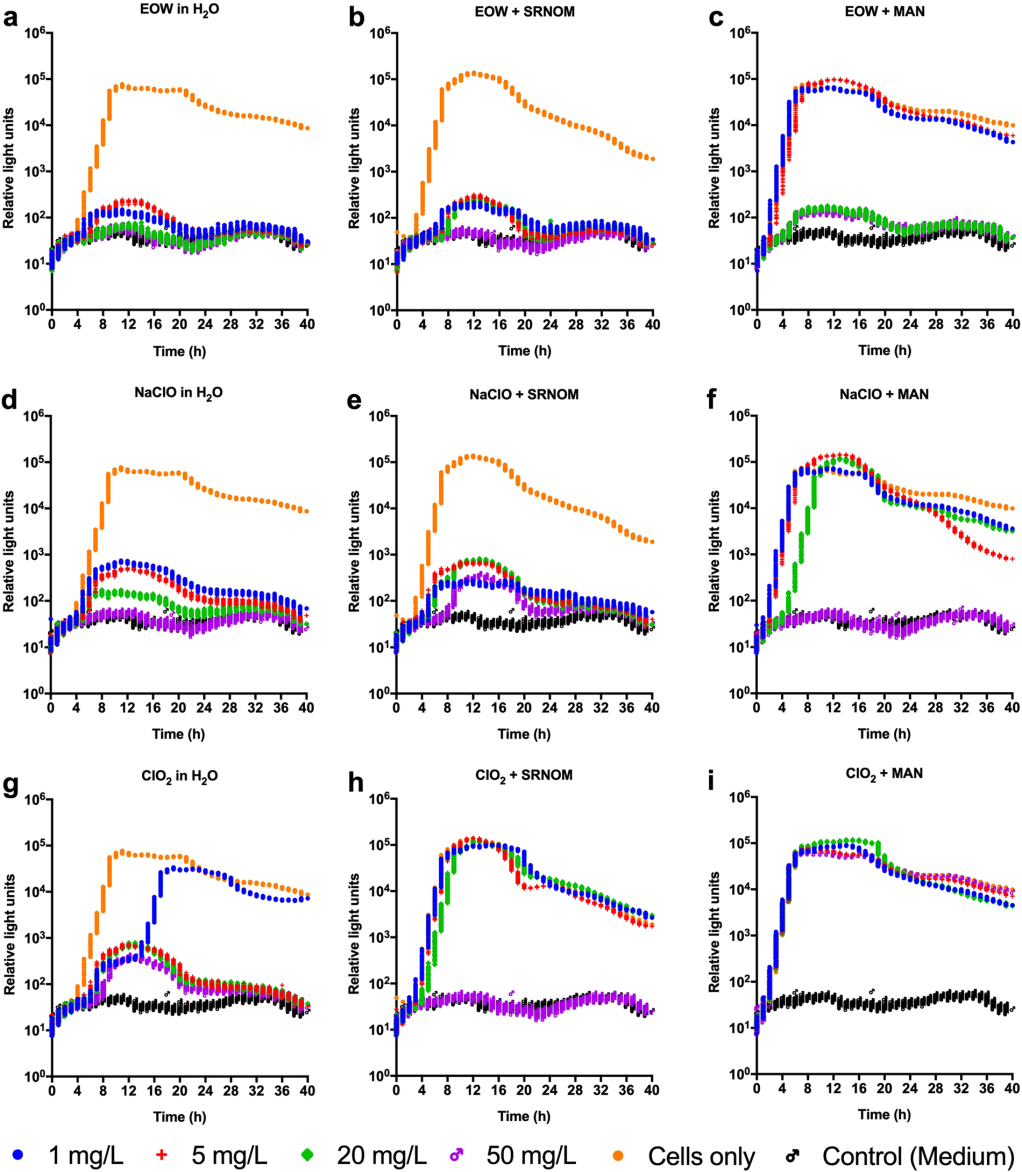
Metabolic activity measurements of bioluminescent *Escherichia coli* Xen14 treated with sanitizers added to artificially-contaminated water. Bacteria were treated with electrolyzed oxidizing water (EOW), sodium hypochlorite (NaClO) or chlorine dioxide (ClO_2_) prepared at 0, 1, 5, 20 and 50 mg/L free chlorine in the presence of either 40 mg/L dissolved organic carbon (DOC) from Suwannee river natural organic matter (SRNOM) or 100 mg/L DOC from cow manure for 120 s. Untreated bacteria resuspended in sterile water, 40 mg/L DOC from SRNOM or 100 mg/L DOC from cow manure were used as controls. Aliquots of treated samples were added to 200 µL sterile Luria Bertani broth and then incubated at 37 °C in a Cytation 5 Cell Imaging Multi-Mode Reader. Total luminescent signals (relative light units) were collected over a 40 h incubation period. Figures were generated using Prism v8 (GraphPad Software, San Diego, CA, USA).

It has been widely reported in the literature that chlorine-based sanitizers have the propensity to induce the VBNC state in bacteria^25,27–29,40–42^. Despite the widespread use of chlorine-based sanitizers, testing for VBNC is not widely undertaken and effective concentrations for disinfection of irrigation or process wash water without induction of VBNC have not been established. The use of sanitizers at concentrations below the effective concentration could result in dissemination or transfer of VBNC cells, which can then resuscitate and lead to outbreaks of food-borne disease^25^. This is of particular concern in low-quality irrigation waters, where disinfection efficacy might be compromised by organic matter content or other factors. Therefore, it was of interest to investigate whether EOW also induces the VBNC state in the bacterial populations being tested. For this assessment, we initially examined the effects of different concentrations of EOW, NaClO or ClO_2_ in the presence or absence of SRNOM or γ-sterilized cow manure on the viability and metabolic activity of bioluminescent *E. coli* Xen14. We found that, in the absence of organic matter, EOW and NaClO were bactericidal (and little to no metabolic activity was observed) in the range of concentrations (1–50 mg/L free chlorine) used over the 40 h incubation period (Fig. 5a,d). However, detectable metabolic activity was already observed for Xen14 treated with ClO_2_ at as low as 1 mg/L free chlorine (Fig. 5g) despite the absence of visible growth on culture plates at this concentration, a strong indication of VBNC bacteria. Furthermore, we found that no metabolic activity was observed for Xen14 treated in the range of EOW and NaClO concentrations at which no growth was observed on agar plates used in the presence of SRNOM or cow manure over the 40 h incubation period (Fig. 5b,c,e). The efficacy of EOW was slightly superior to that of NaClO under these conditions, being bactericidal at 20 mg/L free chlorine, while NaClO was only bactericidal at 50 mg/L free chlorine in the presence of cow manure. The efficacy of ClO_2_ was poor in the presence of organic matter, with complete kill only observed when it was used at the highest concentration (50 mg/L free chlorine) in the presence of SRNOM. Furthermore, ClO_2_ exerted no measurable activity at this concentration in the presence of cow manure. Bacterial plate counts from all treatments were also determined for comparison (Table S1).

**Figure 6:**
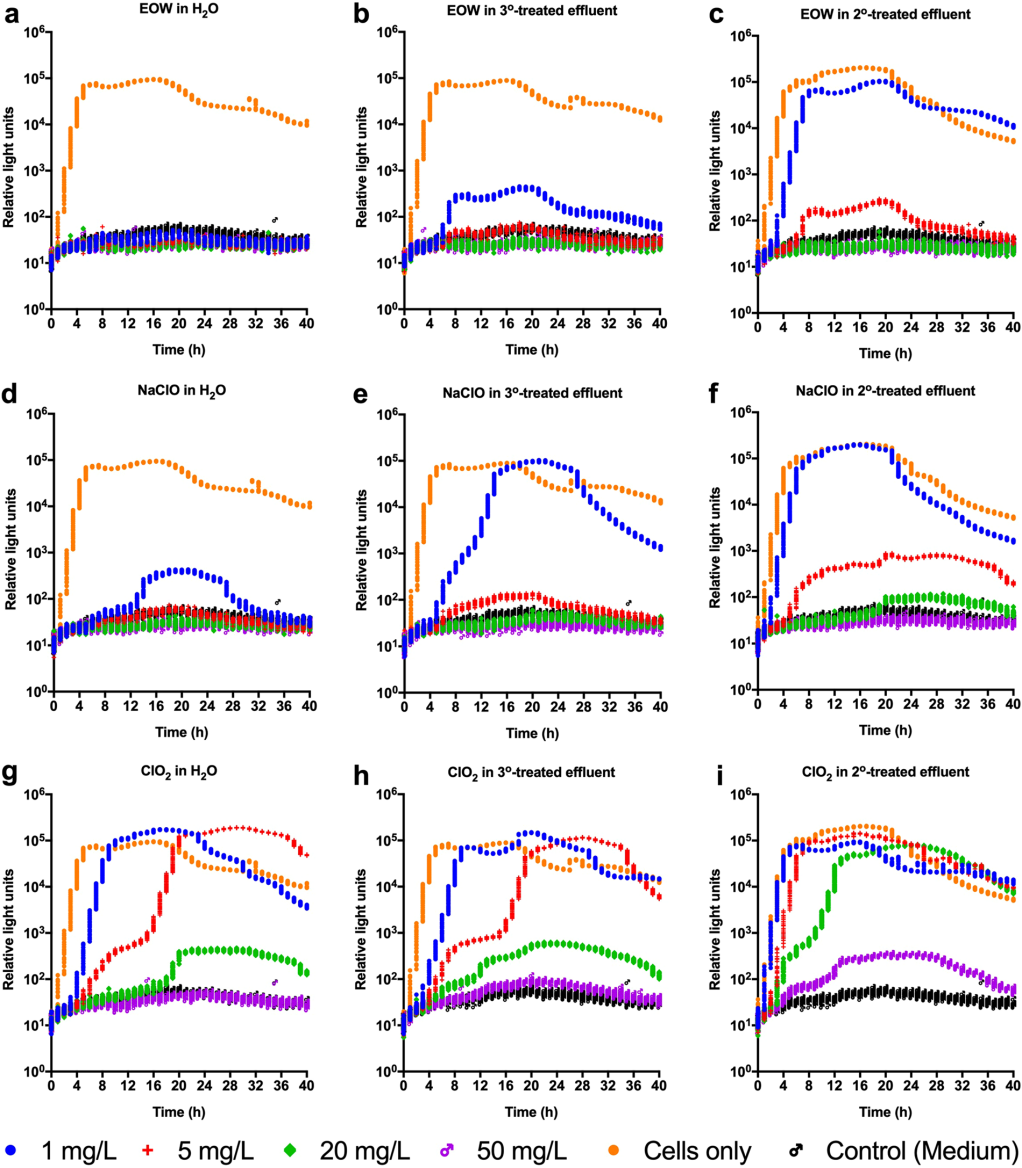
Metabolic activity measurements of bioluminescent *Escherichia coli* Xen14 treated with sanitizers added to wastewater effluent. Bacteria were treated with electrolyzed oxidizing water (EOW), sodium hypochlorite (NaClO) or chlorine dioxide (ClO_2_) prepared at 0, 1, 5, 20 and 50 mg/L free chlorine in the presence of either tertiary (3°)-treated effluent water (containing 5.6 mg/L dissolved organic carbon [DOC]) or secondary (2°)-treated effluent water (containing 19.2 mg/L DOC) for 120 s. Untreated bacteria resuspended in sterile water, 3°-treated or 2°-treated water were used as controls. Aliquots of treated samples were added to 200 µL sterile Luria Bertani broth and then incubated at 37 °C in a Cytation 5 Cell Imaging Multi-Mode Reader. Total luminescent signals (relative light units) were collected over a 40 h incubation period. Figures were generated using Prism v8 (GraphPad Software, San Diego, CA, USA).

There are no equivalent studies for a comparison with the results of this study in terms of DOC as a measure of organic load. Han *et al*.^23^ obtained an effective EOW concentration of 37 mg/L of free chlorine against suspensions of *E. coli*, *S*. Enteriditis and *Yersinia enterocolitica* in the absence of organic matter. A recent study by Afari *et al*.^39^ showed the induction of VBNC *E. coli* and *L. monocytogenes* after treatment of inoculated lettuce wash water with acidic EO water; their results suggested the effective concentration of 9 mg/L acidic EO water in lettuce wash water. However, they used UV_254_ as a measure of the organic load, making it difficult to directly compare to the results of our study. To confirm the above findings using naturally-occurring DOC on sanitizer efficacy, another set of experiments using sterile RO water, tertiary treated wastewater effluent (containing 5.6 mg/L DOC), or secondary treated wastewater effluent (containing 19.2 mg/L DOC) was performed. Again, the efficacy of 1 mg/L EOW was slightly superior to the equivalent concentration of NaClO in RO water and tertiary-treated effluent water (Fig. 6a,b,d,e). The results also show the superior antibacterial efficacy of EOW and NaClO over ClO_2_ under all the conditions tested, but particularly so in the secondary treated wastewater with high DOC content (Fig. 6c,f,i).

**Figure 7:**
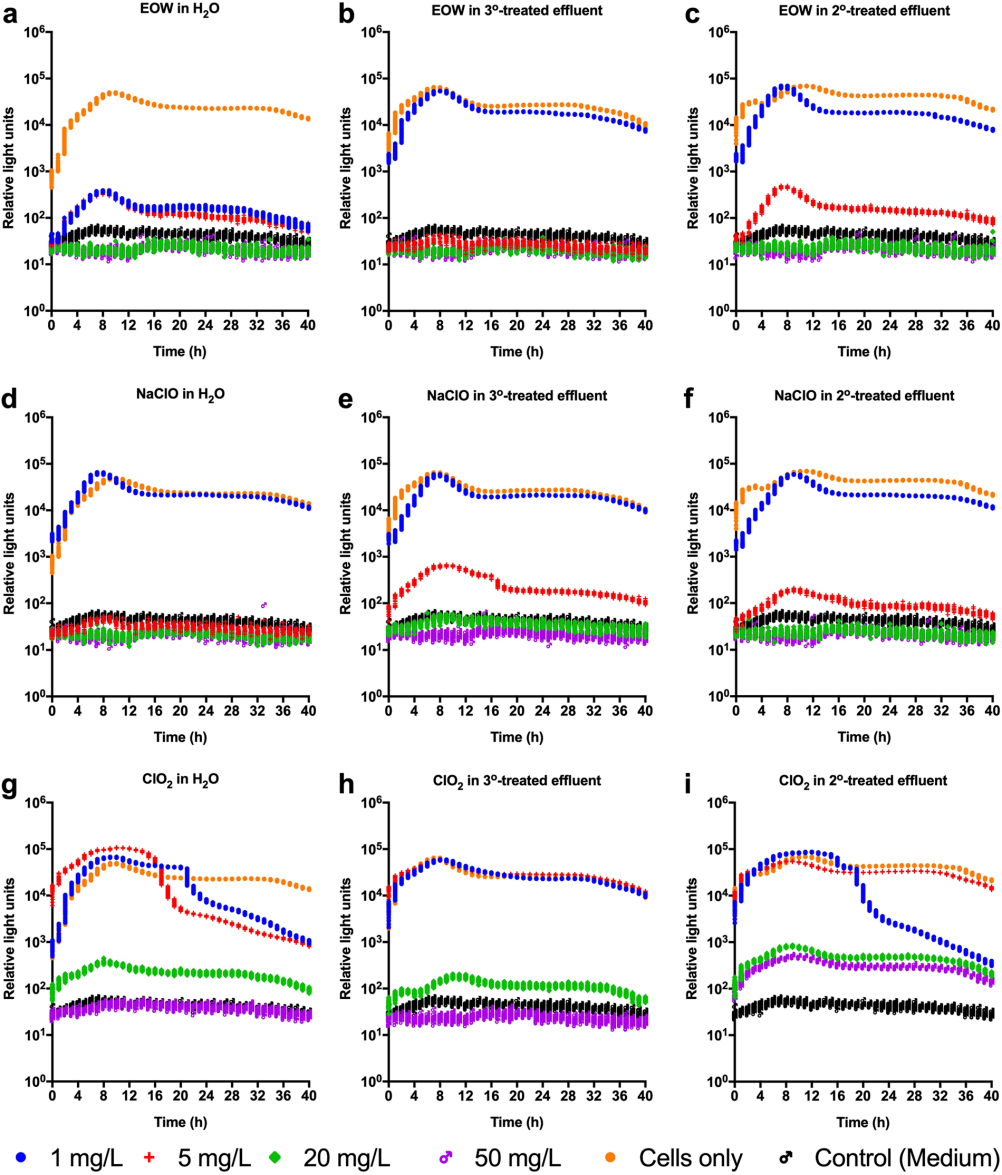
Regrowth of sanitizer-treated bioluminescent *Escherichia coli* Xen14. Aliquots of all samples treated with electrolyzed oxidizing water (EOW) sodium hypochlorite (NaClO) or chlorine dioxide (ClO_2_) in Fig. 6 were added to fresh Luria Bertani broth and then incubated at 37 °C in a Cytation 5 Cell Imaging Multi-Mode Reader for another 40 h. Total luminescent signals (relative light units) were collected over a 40 h incubation period. 3°, tertiary treated effluent water; 2°, secondary treated effluent water. Figures were generated using Prism v8 (GraphPad Software, San Diego, CA, USA).

In a subsequent experiment where aliquots of samples analyzed in Fig. 6 were re-inoculated into fresh LB broth and re-incubated for an additional 40 h to examine the potential for regrowth, the results showed essentially similar trends to those of the initial 40 h incubation (Fig. 7). The results showed that at the effective concentration of the disinfectant, no metabolic activity was detected, indicating effectiveness of the disinfectant. VBNC cells were also undetectable under these conditions. Together with corresponding optical density (*A*_600nm_) measurements (Fig. S1) these data corroborate our postulation that EOW does not appear to induce the VBNC state at its effective concentration.

**Figure 8:**
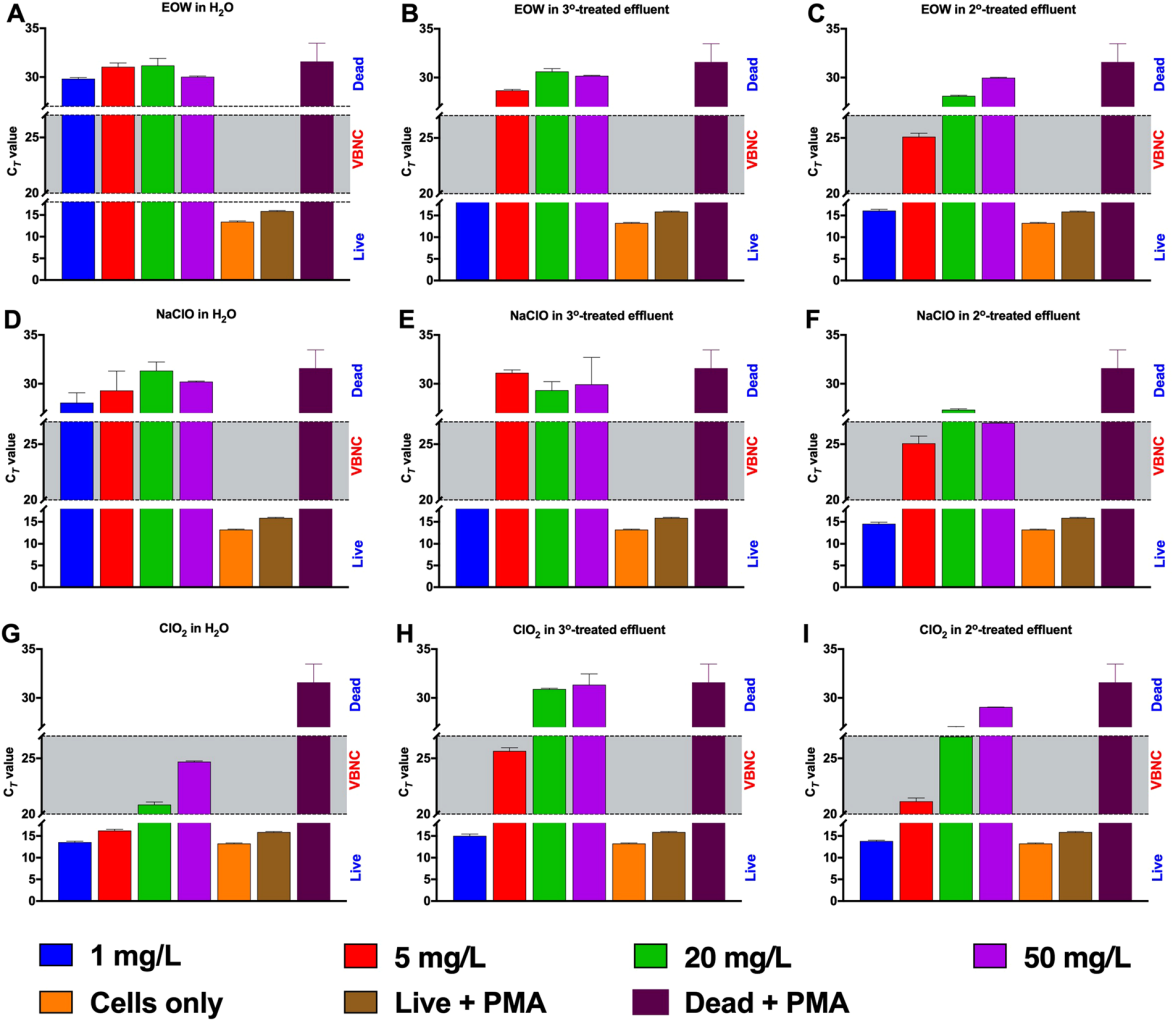
Generation of potential VBNC state in *Escherichia coli* Xen14. Bacteria were treated with various concentrations of sanitizers in the presence of different levels of dissolved organic matter content, after which propidium monoazide (PMAxx) was added. PMAxx is a membrane-impermeable dye that only penetrates and binds to DNA of damaged cells, preventing subsequent PCR amplification. As such, the cycle threshold (C_*T*_) value of intact (live) cells are low (up to 19 C_*T*_); values for potential viable but nonculturable (VBNC) cells range between 20 and 27 C_*T*_, while values for dead cells are from 28 C_*T*_ upwards. For further explanation please see Tenzin *et al*.^32^. Figures were generated using Prism v8 (GraphPad Software, San Diego, CA, USA).

To strengthen the results obtained in the metabolic activity assays, aliquots of the *E. coli* Xen14 cells treated with the various concentrations of EOW, NaClO or ClO_2_ in the presence of sterile water, tertiary-treated effluent water or secondary-treated effluent water described above were treated with the photoreactive DNA binding dye PMAxx™, followed by real-time qPCR analysis. The results largely agree with the metabolic activity assay results, indicating overall superior antibacterial efficacy of EOW and NaClO over ClO_2_ especially in RO water and tertiary-treated wastewater containing 5.6 mg/L DOC content (Fig. 8). Viable counts of bacteria from all treatments were also determined for comparison (Table S2).

## Conclusions

In this study, we have shown that EOW prepared using either Na or K salts significantly reduced the microbial load in artificially contaminated water and demonstrated that its efficacy was not affected under a range of pH conditions but rather by the organic matter content of the water. Furthermore, we showed that the efficacy of EOW to reduce the microbial load was comparable, and in some cases better than that of the other chlorine-based sanitizers (NaClO and ClO_2_). Critically, we showed that at its effective concentration (20 mg/L), EOW did not induce VBNC cells of the surrogate bacterial pathogens tested. In comparison, the effective concentration of NaClO was 50 mg/L and ClO_2_ was not effective at the highest concentration tested. The propensity for ClO_2_ to induce the VBNC state in *E. coli* and other bacteria has been described^25,28,42–44^; the finding that EOW at its effective concentration in the presence of high organic matter did not induce the VBNC state is an additional feature that growers could find advantageous over the use of other chlorine-based sanitizers. To the best of our knowledge, this study is the first to systematically address the effect of organic matter content in terms of DOC on the efficacy of chlorine-based sanitizers, thus providing a benchmark for future studies and application in the field. With these results in mind, we suggest EOW has a strong potential for decontamination of microbiologically-impaired waters for irrigation of fruits and vegetables and/or for post-harvest sanitation of minimally processed fresh produce.

## Supplementary information

Supplementary information

## Data Availability

The datasets generated during and/or analysed during the current study are available from the corresponding author on reasonable request.
