# Supplementary material for: Comparative antibacterial activities of neutral electrolyzed oxidizing water and other chlorine-based sanitizers
Source: Sci Rep. 2019 Dec 27;9:19955. doi: 10.1038/s41598-019-56248-7 (PMC6934530; doi:10.1038/s41598-019-56248-7)
Supplement: Supplementary file 1 — Supplementary information [file 41598_2019_56248_MOESM1_ESM.docx]

**Comparative antibacterial activities of neutral electrolyzed oxidizing water and other chlorine-based sanitizers**

**Abiodun D. Ogunniyi^1^*, Catherine E. Dandie^1^, Sergio Ferro^2^, Barbara Hall^3^, Barbara Drigo^1^, Gianluca Brunetti^1^, Henrietta Venter^4^, Baden Myers^5^, Permal Deo^4^, Erica Donner^1^ and Enzo Lombi^1^**

^1^ Future Industries Institute, University of South Australia, Mawson Lakes, South Australia, Australia

^2^ Ecas4 Australia Pty Ltd, 8/1 London Road, Mile End South, South Australia, Australia

^3^ Plant Health and Biosecurity, SARDI, Adelaide, South Australia, Australia

^4^ School of Pharmacy and Medical Sciences, University of South Australia, Adelaide, South Australia, Australia

^5^ Natural and Built Environments Research Centre, School of Natural and Built Environments, University of South Australia, Mawson Lakes, South Australia, Australia

*Corresponding Author: [david.ogunniyi@unisa.edu.au](mailto:david.ogunniyi@unisa.edu.au), Future Industries Institute, Building X, Mawson Lakes Campus, Mawson Lakes Boulevard, Mawson Lakes, 5095, South Australia, Australia.

**Table S1.** *Escherichia coli* (Xen14) recovery (colony-forming units per mL) after treatment with different concentrations of chlorine-based sanitizers added to artificially-contaminated water.

| **Sanitizer concentration** | **Resuspension solution** | | | | | | | | |
| --- | --- | --- | --- | --- | --- | --- | --- | --- | --- |
|  | **RO water *^a^*** | | | **SRNOM *^a^*** | | | **Cow Manure *^a^*** | | |
|  | **EOW** | **NaClO** | **ClO_2_** | **EOW** | **NaClO** | **ClO_2_** | **EOW** | **NaClO** | **ClO_2_** |
| **1 mg/L** | NGD*^b^* | NGD | NGD | NGD | NGD | 4.20 × 10^6^ | 2.18 × 10^7^ | 1.46 × 10^7^ | 1.98 × 10^7^ |
| **5 mg/L** | NGD | NGD | NGD | NGD | NGD | 1.96 × 10^6^ | 9.60 × 10^6^ | 4.0 × 10^6^ | 1.92 × 10^7^ |
| **20 mg/L** | NGD | NGD | NGD | NGD | NGD | 2.18 × 10^5^ | NGD | 4.20 × 10^5^ | 1.68 × 10^7^ |
| **50 mg/L** | NGD | NGD | NGD | NGD | NGD | NGD | NGD | NGD | 1.64 × 10^7^ |

*^a^* The starting bacterial inoculum in each of these solutions was approx. 2.0 × 10^7^ colony-forming units per mL.

*^b^* NGD= No growth detected (Limit of detection = 40 colony-forming units per mL).

**Table S2.** *Escherichia coli* (Xen14) recovery (colony-forming units per ml) after treatment with different concentrations of chlorine-based sanitizers added to wastewater effluent.

| **Sanitizer concentration** | **Resuspension solution** | | | | | | | | |
| --- | --- | --- | --- | --- | --- | --- | --- | --- | --- |
|  | **RO water *^a^*** | | | **3^o^-treated effluent *^a^*** | | | **2^o^-treated effluent *^a^*** | | |
|  | **EOW** | **NaClO** | **ClO_2_** | **EOW** | **NaClO** | **ClO_2_** | **EOW** | **NaClO** | **ClO_2_** |
| **1 mg/L** | NGD*^b^* | 1.13 × 10^4^ | 2.15 × 10^8^ | 9.25 × 10^4^ | 6.75 × 10^4^ | 2.70 × 10^8^ | 1.90 × 10^8^ | 2.23 × 10^8^ | 2.68 × 10^8^ |
| **5 mg/L** | NGD | NGD | 3.75 × 10^3^ | NGD | NGD | 4.50 × 10^3^ | NGD | 8.0 × 10^1^ | 1.80 × 10^7^ |
| **20 mg/L** | NGD | NGD | NGD | NGD | NGD | NGD | NGD | NGD | 1.25 × 10^3^ |
| **50 mg/L** | NGD | NGD | NGD | NGD | NGD | NGD | NGD | NGD | NGD |

*^a^* The starting bacterial inoculum in each of these solutions was approx. 3.25 × 10^8^ colony-forming units per mL

*^b^* NGD= No growth detected (Limit of detection = 40 colony-forming units per mL).


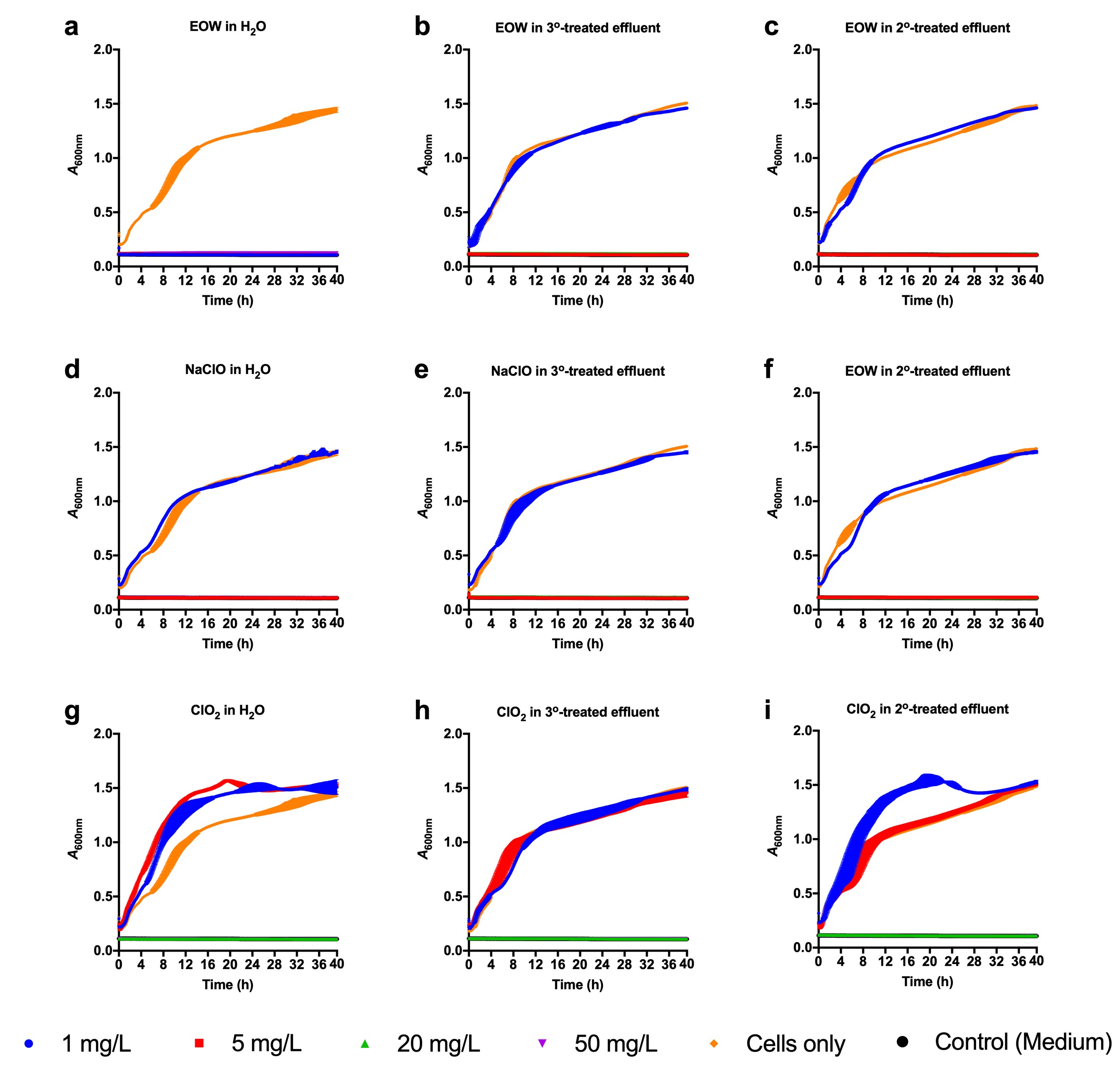


**Figure S1. Optical density measurements of** **sanitizer-treated bioluminescent *Escherichia coli* Xen14**. Aliquots of all samples treated with electrolyzed oxidizing water (EOW) sodium hypochlorite (NaClO) or chlorine dioxide (ClO_2_) in Fig. 6 were added to fresh Luria Bertani broth and then incubated at 37°C in a Cytation 5 Cell Imaging Multi-Mode Reader for another 40 h. Optical density measurements (*A*_600nm_) were collected over a 40 h incubation period. 3^o^, tertiary treated effluent water; 2^o^, secondary treated effluent water. Figures were generated using Prism v8 (GraphPad Software, San Diego, CA, USA).
